# Glandular Odontogenic Cyst in the Anterior Mandible: A Case Report of a Conservative Approach and a Recurrence Detection

**DOI:** 10.3390/diagnostics13081452

**Published:** 2023-04-18

**Authors:** Wladimir Gushiken de Campos, Rita Araújo, Victor Martin, Marília Trierveiler, Pedro Gomes, Celso Augusto Lemos

**Affiliations:** 1 Department of Stomatology, School of Dentistry, University of Sao Paulo, São Paulo 05508-000, Brazil; 2 Laboratory for Bone Metabolism and Regeneration, Faculty of Dental Medicine, University of Porto, 4200-393 Porto, Portugal; 3 LAQV/REQUIMTE, University of Porto, 4100-007 Porto, Portugal; 4 Division of Oral and Maxillofacial Pathology, School of Dentistry, University of Sao Paulo, São Paulo 05508-000, Brazil; 5 Department of Oral Medicine, Faculdade de Odontologia, University of Sao Paulo, São Paulo 05508-000, Brazil

**Keywords:** pathology, oral surgery, jaw cysts, glandular odontogenic cyst

## Abstract

Purpose: The glandular odontogenic cyst (GOC) is considered a rare developmental cyst, with an odontogenic origin and both epithelial and glandular characteristics, with less than 200 reported cases in the literature. Methods: In the present case, a 29-year-old man was referred for evaluation of an asymptomatic slow-growing swelling in the anterior region of the mandible, with one-year history. The patient’s medical history did not reveal any systemic alteration. The extraoral examination did not show enlargement of the facial contour and the intraoral examination showed vestibular and lingual swelling. Panoramic radiography and CT scan revealed a well-defined unilocular radiolucent lesion involving the inferior incisors and canines bilaterally. Results: Histopathological analysis revealed multiple cysts lined by stratified epithelium with varying thickness and characteristics, in addition to duct-like structures filled with PAS-positive amorphous material, suggestive of GOC. Conservative treatment was performed through surgical curettage, peripheral ostectomy of the surgical site and apicectomy of the teeth involved in the lesion. There was one recurrence, which was detected in postoperative follow-up, leading to a new surgical approach. Conclusions: Fifteen months after the second procedure, no signs of recurrence were identified, and bone neoformation within the surgical site occurred, supporting that a conservative approach for the treatment of GOC is viable.

## 1. Introduction

The glandular odontogenic cyst (GOC) is a rare odontogenic cystic lesion of the jaws, defined as “a cyst arising in the tooth-bearing areas of the jaws and characterized by an epithelial lining, with cuboidal or columnar cells, both at the surface and lining crypts or cyst-like spaces within the thickness of the epithelium” [1]. 

It is a relatively new entity as it was first described in 1987 by Padayachee and Van Wyk [2]. It was first added to the World Health Organization’s (WHO) odontogenic tumors classification in 1992, with the designation of ”glandular odontogenic cyst” or “sialo-odontogenic cyst” [1]. The last WHO classification, in 2017 [3], classifies the GOC as a developmental cyst with epithelial features that simulate salivary gland or glandular differentiation. GOC accounts for approx. 0.4% of all odontogenic cysts and its etiology is presently unknown. It is assumed that the GOC is a lesion originating from remnants of the dental lamina [4,5,6].

Chrcanovic and Gomez [7] conducted a review of all published cases of GOC. Among 169 cases, they found that the lesion was slightly more prevalent in men 1.15:1, with the highest prevalence in the 5th and 6th decades of life, commonly occurring in the anterior region of the mandible. Most lesions present a unilocular radiographic appearance (61.5%), commonly related to bone expansion (73%) [7], cortical perforation, teeth displacements, and even root resorption [6]. GOC has a higher incidence in the mandible (21%), specifically in the anterior region [6]. At early stages, these cysts are often asymptomatic, while large lesions frequently produce symptoms [8]. Due to this characteristic, it has little association with unerupted teeth, since most lesions are located far from the third molar, which is the case for the majority of unerupted teeth associated with lesions [9]. However, more than 20 cases were reported as radiolucent lesions with a dentigerous relationship, underlining the lack of knowledge regarding this cyst [10]. In addition, other concomitant lesions were reported with GOC, namely odontoma and florid cemento-osseous dysplasia, hindering the diagnosis and adding complexity to the treatment [8,11]. Maruyama et al. described one GOC lesion that was transformed into central mucoepidermoid carcinoma, a lesion with an even higher recurrence rate that demands a more aggressive treatment approach [12]. One hypothesis about these conjunctions is their developmental origins. Moreover, due to its common location and radiographic appearance, aneurysmal bone cyst, mucoepidermoid carcinoma, ameloblastoma and odontogenic keratocyst can be included as differential diagnosis of GOC [13].

Although GOC is a recently described lesion, its recognition and definitive diagnosis are essential due to its potential for locally aggressive behavior and a tendency to recur [9]. Treatment performed may vary between conservative approaches, such as curettage/excision and enucleation, and radical approaches, such as mandibular resection [7].

Management guidelines in the literature have been based on case reports, not scientific evidence. This absence of a technical basis is expected (it is highly complex to carry out a study with a robust methodology) since GOC is a very rare lesion [14].

Herein, a case of recurrent GOC is presented along with its differential diagnosis, histopathological features and treatment—all of which are emphasized and discussed.

## 2. Case Report

A 29-year-old male patient was referred to the Stomatology Clinic, School of Dentistry of the University of São Paulo, with an asymptomatic slow-growing swelling in the anterior region of the mandible, for approximately one year. The patient’s medical history did not reveal any systemic alteration.

**Figure 1:**
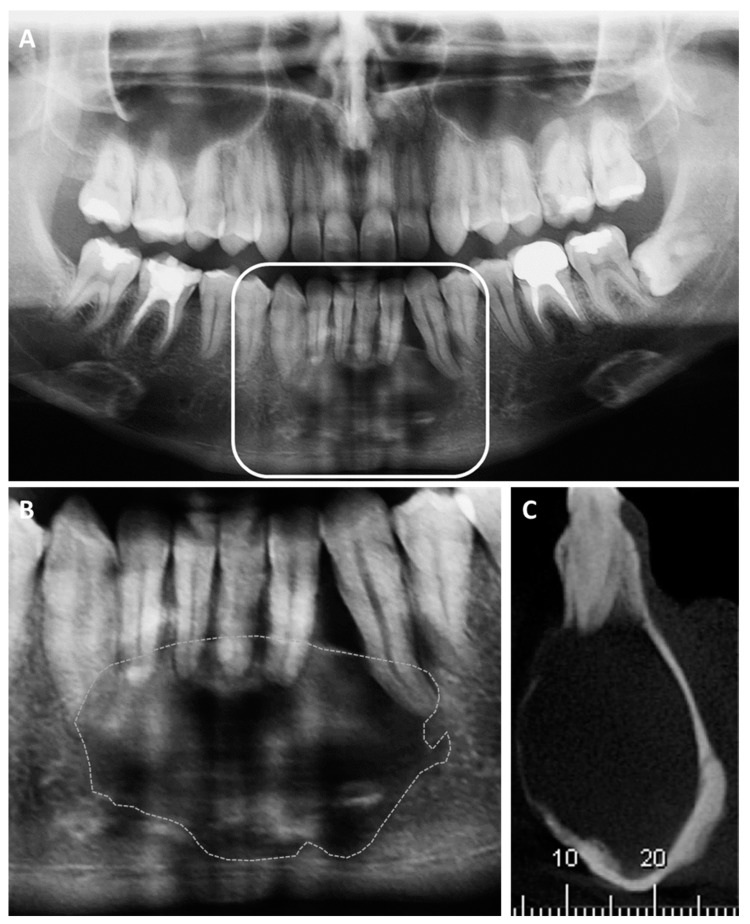
(**A**,**B**)—Initial panoramic radiography, revealing a well-defined unilocular radiolucent lesion between inferior canines, reaching the mandibular base. The dashed line highlights the limits of the lesion. (**C**)—Representative sagittal cut of the preoperative CT scan, confirming a significant radiolucent lesion involving the vestibular and lingual regions.

The extraoral examination did not show enlargement of the facial contour, and the intraoral examination showed vestibular swelling between the mandibular canines, in addition to a slight bulge in the lingual region, with healthy mucosa and no signs of inflammation. Panoramic radiography revealed a well-defined unilocular radiolucent lesion involving the mandibular incisors and canines bilaterally, reaching the mandibular base (Figure 1). In addition, the root of the left inferior canine appeared to be slightly displaced, suggesting a lesion with a high growth rate. Diagnostic hypotheses included central giant cell granuloma and ameloblastoma, substantiating the need for an incisional biopsy, which was performed under local anesthesia, after signature of the informed consent by the patient. Moreover, the patient was referred for a CT scan for a precise diagnosis, determining its borders and anatomical relation with vascular and nervous structures, in order to establish an appropriate surgical plan.

**Figure 2:**
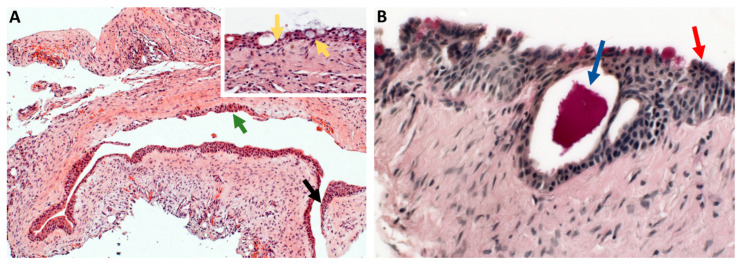
Photomicrographs of the histopathological analysis, detailing the morphological features. (**A**)—Hematoxylin and eosin (H&E) staining: Thin connective tissue capsule lined by stratified epithelium that is partly squamous (black arrow), partly cuboidal (green arrow). The epithelium also presented areas of thickening and papillary projections towards the lumen (100× in the original). Inset shows the presence of mucous cells and duct-like structures (yellow arrows) (400×). (**B**)—PAS-diastase staining evidenced hobnail cells (red arrow) on the epithelial surface and mucin-filled intra-epithelial duct-like structures (blue arrow).

The histopathological examination showed multiple cysts lined by stratified epithelium with varying thicknesses and characteristics. The cystic lining was characterized partially by a flat squamous stratified epithelium with areas of thickening and partially by a cuboidal epithelium, evidencing foci of hobnail cells. Clear cells and mucous cells were present, in addition to duct-like structures, which were filled with eosinophilic amorphous material that was positive for periodic acid–Schiff (PAS) staining after diastase digestion. The cyst wall was composed of dense connective tissue and a mild diffuse chronic inflammatory infiltrate (Figure 2). Based on these characteristics, the diagnosis of GOC was established.

A conservative surgical approach was planned, which consisted of curettage and peripheral ostectomy of the surgical site and apicectomy of the teeth involved in the lesion. Preoperatively, the patient was referred to an endodontist for root canal treatment of the involved teeth—the mandibular central and lateral incisors and canines, bilaterally.

**Figure 3:**
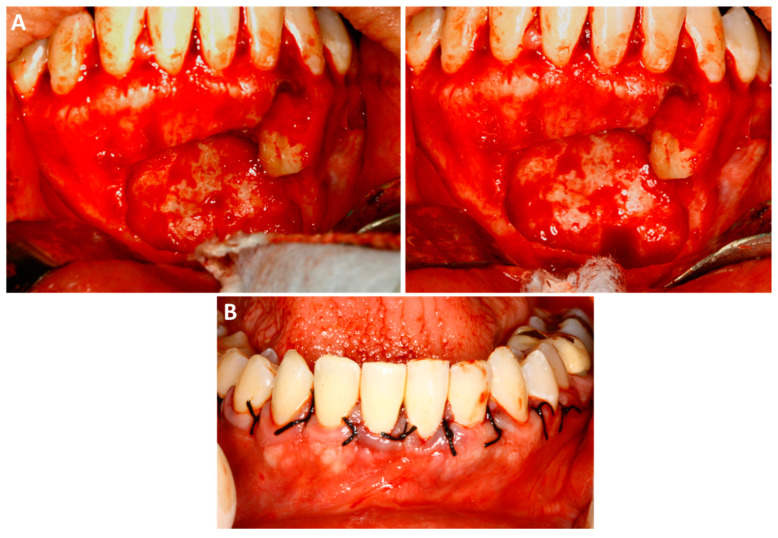
Trans-operative images of the surgical procedure. (**A**)—First surgery—the surgical access and the ostectomy were performed via vestibular cortex. (**B**)—Surgical flap was positioned, and sutures were performed.

The removal of the lesion was performed under local anesthesia, upon the administration of three anesthetic cartridges of 2% mepivacaine with epinephrine 1:100,000 (Mepiadre, DFL, 1.8 mL, Brazil). An osteotomy was performed in the vestibular cortex to access the lesion (Figure 3A). Following this, after careful curettage, an apicectomy of the involved teeth was performed to access the posterior region of the roots in order to allow a proper peripheral ostectomy at this region of the surgical site. Careful root scaling and planning of the involved teeth were also performed. The closure was done using interrupted sutures (Figure 3B). The entire removed lesion was sent for histopathological examination, which confirmed the diagnosis of GOC.

**Figure 4:**
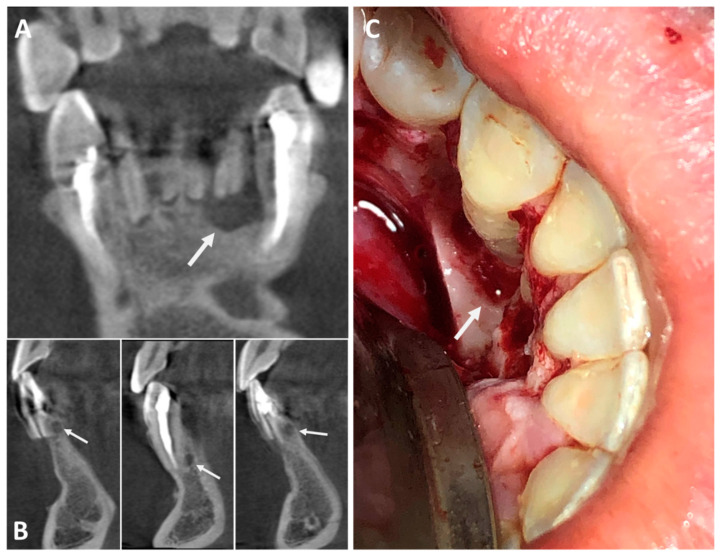
(**A**)—Representative coronal cut and (**B**)—sagittal cuts of the CT scan 8 months after the first surgery, revealing recurrent lesions in the apical region, lingually. (**C**)—Trans-operative image of the second surgical approach, aiming the removal of the recurrent lesion in the lingual portion of the cortex. The arrows point to the recurrent lesions.

Considering the high recurrence rates of GOC, adequate follow-up appointments were set. Eight months after the first procedure, a recurrence was detected by CT scan in the lingual portion of the cortical bone (Figure 4A,B). The patient underwent a new surgical approach under local anesthesia. The lesion was accessed through an incision at the lingual region—osteotomy and curettage were then performed, as previously described (Figure 4C).

**Figure 5:**
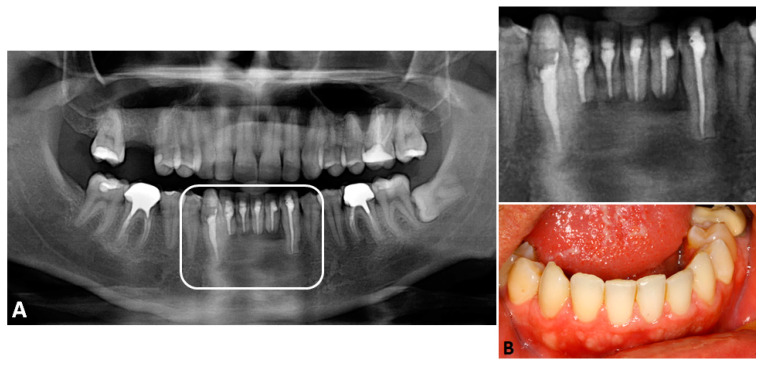
Follow-up of fifteen months after the second procedure. (**A**)—The panoramic radiograph showed bone neoformation within the entire surgical site, with no signs of recurrence. (**B**)—Clinically, the mucosa was found to be healthy, and the involved teeth were in function, without functional or aesthetic repercussions.

Fifteen months after the second procedure, the panoramic radiograph showed a nearly complete bone neoformation within the surgical site. Clinically, the mucous membranes were found to be intact, with no signs of recurrence. Furthermore, all teeth were in function, without clinical or aesthetic repercussions (Figure 5).

## 3. Discussion

The GOC is a rare lesion of the jaws that affects mainly men between the 5th and 6th decades of life, with reported frequency rates ranging from 0.012% to 1.3% [7,15,16,17]. Despite being first described in the 1980s, its odontogenic origin was only confirmed in 2011 by immunohistochemical methods and its pathophysiology is yet to be understood [18]. Small GOC cysts are generally asymptomatic, while sizeable lesions can cause pain or paresthesia [18]. In the reported case, the patient was a 29-year-old male; the affected region was the anterior mandible, with bone expansion and a radiolucent radiographic image, which is in line with most cases described in the literature.

Regarding dental changes, GOC seems to be related to an unerupted tooth or tooth displacement in 30.9% of cases, root resorption in 13.8%, cortical bone perforation in 26% and clinical symptomatology in 24.3% [7]. In our reported case, no tooth displacement, root resorption or clinical symptomatology were present. Swelling is the first symptom of GOCs in 88% of cases, while 9% are detected as incidental findings during routine radiographic examination [9]. Similarly, in the present case, the lesion was only detected after the beginning of the bone expansion, as the patient had no other symptoms and only a minor root displacement could be presumed by the radiography.

GOC does not have a pathognomonic radiological appearance. Several lesions of the jaws, such as low-grade mucoepidermoid carcinoma, central giant cell lesion, odontogenic keratocyst, ameloblastoma, and botryoid odontogenic cyst, have similar radiological characteristics, being common diagnostic hypotheses [19]. Moreover, despite being uncommon in the anterior region of the jaw, Stafne bone cavity can have an analogous radiological appearance to GOC [20]. However, this defect generally presents a bone depression in the lingual surface rather than swellings, being easily distinguished from tumors with three-dimensional imaging [21]. Due to the higher recurrence rates of GOC in comparison with all these lesions, having the correct diagnosis is of the utmost importance [8].

In a systematic review that analyzed radiological features of GOC, lesions were unilocular in 29 patients and multilocular in 27 (48%). In addition, these lesions had a width ranging from 0.5 to 12 cm (mean 4.9 cm), most of them had cortical expansion, comprising 87% (33/38), cortical perforation in 50% (12/24) and presented tooth root resorption in 22% (9/41) and tooth displacement in 24.4% (10/41) [22]. Its high cellular proliferation rate is mostly related to an augmented Ki-67 index and dysregulation of cell death, caused by an abnormal presence of anti-apoptotic proteins, such as B-cell lymphoma 2 (Bcl-2) [23].

Perhaps the most controversial topic about the GOC is the histological criteria to confirm the diagnosis, without an established consensus in the literature. GOC histological features may be similar to many oral lesions, such as radicular, dentigerous, botryoid cysts or mucoepidermoid carcinoma [17]. Moreover, due to unclear diagnostic criteria, the histological identification of GOC is problematic. Several distinct histological features are described in reported cases and, despite a morphological similarity with salivary glands in the cyst’s lining, which is why the lesion was initially called a sialo-odontogenic cyst, the most recent evidence favors the hypothesis of odontogenic origin [24]. For Fowler et al. (2011) [17], at least five of ten microscopic parameters should be present for a lesion to be confirmed as GOC: (1) eosinophilic cuboidal cells on the surface; (2) intraepithelial microcysts or duct-like spaces lined by a single layer of cuboidal to columnar cells; (3) apocrine snouting of hobnail cells; (4) clear or vacuolated cells; (5) variable thickness of the cyst lining; (6) papillary projections; (7) mucous goblet cells; (8) epithelial spheres or plaque-like thickenings; (9) cilia; (10) multiple compartments. In the reported case, histological analysis scored 9 out of 10 due to the absence of cilia, thus confirming the diagnosis of GOC. Recently, another diagnosis approach was proposed in relation to immunohistochemistry and biomarkers—Maruyama et al. observed that CK13 was exclusively expressed in GOC lesions in comparison to central mucoepidermoid carcinoma, while CK17 expression was related to malignancies [12]. Moreover, Junior et al. [25] compared the expression of three different biomarkers of GOC, odontogenic keratocyst and botryoid odontogenic cyst and found that high expression of epidermal growth factor receptor (EGFR) was related to botryoid odontogenic cysts and GOCs presented higher expression of nuclear Cyclin D1. Furthermore, Kaplan et al. suggest that Ki-67 and p53 biomarkers can help in differentiating GOC from low-grade mucoepidermoid carcinoma [24]. However, it was pointed out that the only exclusive biomarker was SOX2 of odontogenic keratocysts. Nonetheless, studies exploring different biomarkers are a step in the right direction, facilitating and increasing the precision of the diagnosis. The present case report highlights the importance of an adequate incisional biopsy sample, a careful observation of histological cuts, seeking the combination of the relevant findings and serving as an example of a clear case of GOC.

Concerning the GOC treatment, it may be performed through conservative approaches, such as curettage/excision and enucleation, or radical approaches, such as partial resection or resection with continuity. In the study of Chrcanovic and Gomez [7], from the 122 cases, 108 were treated conservatively and 12 were treated aggressively. Among all, 97 cases had follow-up recurrence information, of which 21 recurred (21.6%), 20 cases were treated conservatively, and one was treated aggressively. For the authors, treatment of this lesion must involve adjunctive therapy after excision, such as carnoy’s solution, cryotherapy or peripheral ostectomy to avoid recurrences. Fowler et al. [17] found higher recurrence rates (50%), as their study included 46 cases treated by conservative excisions. In the study by Kaplan et al. [14], the clinical and radiographic characteristics of patients with and without recurrence were analyzed, showing that, the more locular the lesion is, the greater probability of recurrence is expected (64.3% vs. 41.2%). In addition, the size of the lesion is directly related to relapses, with large lesions presenting a recurrence rate of approximately six times higher than smaller cysts [24]. This high reappearance rate of GOC may be associated with its biological characteristics—the thinness of the cyst wall, in addition to a tendency of the epithelium layer to detach from the connective tissue can result in the formation of microcysts and epithelial remains, hindering its complete removal [23,24].

In the present case, after an initial conservative approach, one recurrence occurred, which was promptly treated. After 15 months, no recurrences were detected. Bone neoformation was verified within the entire surgical site and the maintenance of all teeth involved in the lesion was achieved, with no visible scars.

If the treatment had been radical, all teeth involved in the lesion would have been lost, further compromising speech, mastication, and facial aesthetics. In addition, the patient would have experienced significant morbidity since resection of such extension would have required bone reconstruction with a free graft or microsurgical flap. In a recent study [26], the health-related quality of life of patients treated for a locally aggressive odontogenic lesion was assessed. It was observed that after radical surgical resection, most patients presented low scores on masticatory function, as most patients are not rehabilitated following the surgical resection. Despite the prevention of recurrences as the backbone of the treatment planning, it is also paramount to include a swift oral prosthetic rehabilitation to provide the return of a physiologic oral condition. For the above-mentioned, one could argue that conservative treatment should be considered as the first approach, if a close and extended follow-up is feasible, as the need for extensive oral rehabilitation is avoided or further delayed.

## 4. Conclusions

GOC is a rare, locally aggressive odontogenic cyst that, besides with regards to high recurrence rates, may be treated with a conservative approach, thus preserving involving teeth and anatomical structures, and diminishing the morbidity associated with radical surgical treatment. In the present report, one recurrence was detected 8 months after the removal of the lesion, reinforcing the importance of follow-up, especially when a conservative approach is chosen, as in this reported case. Nevertheless, extended monitoring periods are required, regardless of the modality of treatment.

## Data Availability

The data presented in this study are available on request from the corresponding author.

## References

[1] Kramer I.R.H., Pindborg J.J., Shear M. (1992). The WHOHistological typing of odontogenic tumours. A commentary on the second edition. Cancer.

[2] Padayachee A., Wyk C.W. (1987). Two cystic lesions with features of both the botryoid odontogenic cyst and the central mucoepidermoid tumour: Sialo-odontogenic cyst?. J. Oral Pathol. Med..

[3] Wright J.M., Vered M. (2017). Update from the 4th Edition of the World Health Organization Classification of Head and Neck Tumours: Odontogenic and Maxillofacial Bone Tumors. Head Neck Pathol..

[4] Jones A.V., Craig G.T., Franklin C.D. (2006). Range and demographics of odontogenic cysts diagnosed in a UK population over a 30-year period. J. Oral Pathol. Med..

[5] Wright E.F., North S.L. (2009). Management and Treatment of Temporomandibular Disorders: A Clinical Perspective. J. Man. Manip. Ther..

[6] Martins-Chaves R.R., Granucci M., Gomez R.S., Henriques de Castro W. (2021). Glandular Odontogenic Cyst—A Case Series. J. Oral Maxillofac. Surg..

[7] Chrcanovic B.R., Gomez R.S. (2018). Glandular odontogenic cyst: An updated analysis of 169 cases reported in the literature. Oral Dis..

[8] Chen K., Luo C., Qiu J., Zhang Q. (2022). Glandular Odontogenic Cyst Associated with Odontoma: A Rare Case Report. J. Maxillofac. Oral Surg..

[9] MacDonald-Jankowski D.S. (2010). Glandular odontogenic cyst: Systematic review. Dentomaxillofac. Radiol..

[10] Lai P.-T., Li C.-Y., Wu Y.-C., Chiang C.-P. (2022). Glandular odontogenic cyst in a dentigerous relationship. J. Dent. Sci..

[11] Kungoane T., Robinson L. (2021). Florid Cemento-Osseous Dysplasia with a Concurrent Glandular Odontogenic Cyst. Head Neck Pathol..

[12] Maruyama S., Mori T., Yamazaki M., Abé T., Ryo E., Kano H., Hasegawa G., Tanuma J. (2021). Central mucoepidermoid carcinoma arising directly from a glandular odontogenic cyst of the mandible: A case report. Diagn. Pathol..

[13] Senthilmurugan M., Periasamy S., Kumar S.P., Thota R. (2021). Glandular Odontogenic Cyst: A Diagnostic and Management Dilemma. Cureus.

[14] Kaplan I., Gal G., Anavi Y., Manor R., Calderon S. (2005). Glandular odontogenic cyst: Treatment and recurrence. J. Oral Maxillofac. Surg..

[15] Magnusson B., Göransson L., Odesjö B., Gröndahl K., Hirsch J.M. (1997). Glandular odontogenic cyst. Report of seven cases. Dentomaxillofac. Radiol..

[16] Van Heerden W.F.P., Raubenheimer E.J., Turner M.L. (1992). Glandular odontogenic cyst. Head Neck.

[17] Fowler C.B., Brannon R.B., Kessler H.P., Castle J.T., Kahn M.A. (2011). Glandular Odontogenic Cyst: Analysis of 46 Cases with Special Emphasis on Microscopic Criteria for Diagnosis. Head Neck Pathol..

[18] Heiliczer S., Shmuly T., Avishai G., Zlotogorski-Hurvitz A., Vered M., Mamber L., Kaplan I. (2022). Histopathological and histomorphometric analysis of glandular odontogenic cyst-A diagnostic aid. Oral Dis..

[19] Boffano P., Cassarino E., Zavattero E., Campisi P., Garzino-Demo P. (2010). Surgical Treatment of Glandular Odontogenic Cysts. J. Craniofac. Surg..

[20] Bornstein M.M., Wiest R., Balsiger R., Reichart P.A. (2009). Anterior Stafne’s Bone Cavity Mimicking a Periapical Lesion of Endodontic Origin: Report of Two Cases. J. Endod..

[21] Chaweeborisuit P., Yurasakpong L., Kruepunga N., Tubbs R.S., Chaiyamoon A., Suwannakhan A. (2023). The prevalence of Stafne bone cavity: A meta-analysis of 355,890 individuals. J. Dent. Sci..

[22] Manor R., Anavi Y., Kaplan I., Calderon S. (2003). Radiological features of glandular odontogenic cyst. Dentomaxillofac. Radiol..

[23] Krishnamurthy A., Sherlin H.J., Ramalingam K., Natesan A., Premkumar P., Ramani P., Chandrasekar T. (2009). Glandular odontogenic cyst: Report of two cases and review of literature. Head Neck Pathol..

[24] Kaplan I., Anavi Y., Hirshberg A. (2008). Glandular odontogenic cyst: A challenge in diagnosis and treatment. Oral Dis..

[25] Júnior J.F., de França G.M., da Silva Barros C.C., Felix F.A., da Silva W.R., de Lucena H.F., Oliveira C.N., Galvão H.C. (2022). Biomarkers involved in the proliferation of the odontogenic keratocyst, glandular odontogenic cyst and botryoid odontogenic cyst. Oral Maxillofac. Surg..

[26] de Campos W.G., Alkmin Paiva G.L., Esteves C.V., Rocha A.C., Gomes P., Lemos Júnior C.A. (2022). Surgical Treatment of Ameloblastoma: How Does It Impact the Oral Health-Related Quality of Life? A Systematic Review. J. Oral Maxillofac. Surg..

